# Stress and synaptic density in psychosis and clinical high risk: evidence from [^18^F]SynVesT-1 PET

**DOI:** 10.1038/s41398-026-03993-9

**Published:** 2026-04-15

**Authors:** M. Belen Blasco, Kankana Nisha Aji, Christian Ramos-Jiménez, Daniel Chartrand, Chris Hung-Hsin Hsiao, Robert Hopewell, Gassan Massarweh, Johan Cohen, Pablo M. Rusjan, Romina Mizrahi

**Affiliations:** 1 Integrated Program in Neuroscience, McGill University, Montreal, QC Canada; 2 Clinical and Translational Sciences Lab, Douglas Research Centre, Montreal, QC Canada; 3 Department of Anesthesia, McGill University, Montreal, QC Canada; 4 McConnell Brain Imaging Centre, Montreal Neurological Institute, McGill University, Montreal, QC Canada; 5 Douglas Mental Health University Institute, McGill University, Verdun, QC Canada; 6 Department of Psychiatry, McGill University, Montreal, QC Canada

**Keywords:** Schizophrenia, Molecular neuroscience

## Abstract

Synaptic dysfunction is implicated in the pathophysiology of schizophrenia, and positron emission tomography (PET) studies demonstrate in vivo reductions in synaptic density across illness stages. Stress is a key modifiable risk factor, and while animal studies show it disrupts synaptic function, its effects on humans remain unclear. We examined the relationship between stress and synaptic density in individuals with first-episode psychosis (FEP) and those at clinical high risk (CHR). Seventy-eight participants, including 25 FEP, 32 CHR, and 21 healthy controls (HC), underwent 90-min [^18^F]SynVesT-1 PET scans to quantify synaptic density measured as SV2A binding across prioritized brain regions. Stress-related measures included the Hassles and Uplifts Scale and the Trier Inventory for Chronic Stress (TICS). Depressive symptoms were evaluated using the Hamilton Depression Rating Scale (HDRS). Across all participants, greater acute stress was associated with lower [^18^F]SynVesT-1 binding (F_1449_ = 12.0, *p* < 0.001), with no significant group interaction (F_2449_ = 2.44, *p* = 0.09). Group differences emerged for chronic stress and depressive symptoms (TICS × Group: F_2431_ = 3.87, *p* = 0.02; sqHDRS × Group: F_2443_ = 4.47, p = 0.01). Post hoc analyses revealed that higher chronic stress was associated with lower synaptic density in HC (F_1119_ = 7.07, p = 0.009) but not in clinical groups. Lower mood symptoms were associated with lower synaptic density in FEP (F_(1141)_ = 5.19, *p* = 0.02) only. These findings indicate that the relationship between stress and synaptic density differs between clinical and healthy groups. The changes in the relationship between stress and synaptic density in FEP may reflect impaired adaptive neuroplasticity, providing a potential mechanism by which stress contributes to psychosis vulnerability.

## Introduction

Schizophrenia remains a significant public health challenge due to its debilitating effects and lifelong burden [1]. Recent advances suggest that synaptic dysfunction plays a role in its pathophysiology [2]. Evidence from genetic [3], postmortem [4, 5], and in vivo studies [6–10] consistently demonstrates reductions in synaptic elements, highlighting the importance of targeting synaptic integrity as a potential therapeutic venue.

Positron emission tomography (PET) imaging enables in-vivo quantification of pre-synaptic markers. Specifically, radioligands bind to synaptic vesicle glycoprotein 2 A (SV2A), which is ubiquitously expressed on nerve terminals throughout the brain, [11] and highly correlates with gold standard presynaptic markers (e.g., synaptophysin) [12]. To date, PET studies across different stages of schizophrenia spectrum disorders - including two in schizophrenia (duration of illness ~ 17 years) [7, 8], three in early-stage psychosis (duration of illness < 3 years) [6, 9, 10], and one in individuals at clinical high risk (CHR) [9] show lower cortical synaptic density (SV2A), albeit smaller in magnitude for early stage and its putative prodrome. Notably, the study in CHR individuals also revealed deficits in the striatum, possibly related to cannabis use [9], a well-known risk factor for schizophrenia development [13]. To date, the impact of other critical environmental risk factors on synaptic density, such as stress, remains unknown.

Stress is considered a significant, modifiable risk factor for schizophrenia [14]. Individuals with schizophrenia often report heightened perceived stress [15] and a higher prevalence of early-life adversity [16]. Adolescents exposed to chronic daily stressors are more likely to exhibit attenuated psychotic symptoms [17], and elevated cortisol levels correlate with symptom progression in the putative prodrome [18]. Furthermore, impaired stress tolerance has been linked to the emergence of prodromal symptoms [19], increased dopamine release [20], and conversion to psychosis in those at risk, while stressful life events can lead to relapse in first episode psychosis (FEP) [21].

Preclinical and clinical evidence show that stress can impair synaptic function. In rodent models, chronic stress induces microglia-dependent synaptic pruning in the prefrontal cortex, leading to reductions in synaptic branching and spine density [22]. Additionally, chronic stress reduces SV2A expression, as assessed with PET imaging in both male and female rats [23]. These changes are associated with social avoidance and anhedonic behavior [22]. Similarly, an in vivo PET study reported synaptic density deficits associated with depression severity in stress-related disorders [24]. Although these effects have primarily been shown in affective disorder patients, emerging evidence suggests their putative relevance in psychosis. Notably, our previous work demonstrated a relationship between synaptic density and negative symptoms in schizophrenia [9], with anhedonia being a first-rank domain of these symptoms [25]. Furthermore, perceived stress mediates the relationship between early-life adversity and anticipatory anhedonia in CHR and depression groups [26]. Taken together, these findings suggest that stress-induced synaptic alterations may underlie a transdiagnostic construct encompassing psychosis and affective disorders.

Here, we investigated the relationship between regional synaptic density ([^18^F]SynVesT-1 BP_ND_) and stress in recent schizophrenia spectrum disorder patients and CHR individuals compared to healthy controls. To our knowledge, this is the first study aiming to directly investigate the influence of stress on synaptic density using [^18^F]SynVesT-1 PET. We hypothesize that the relationship between stress and synaptic density will differ across study groups. Acute and chronic perceived stress will be associated with lower synaptic density in CHR and FEP participants, but not in healthy controls. Similarly, the severity of depressive symptoms will be related to lower synaptic density in the clinical groups, but not in healthy controls. Moreover, we aim to replicate our prior findings of lower synaptic density in early psychosis and CHR states on a larger clinical sample.

## Materials and methods

### Participants

Participants were recruited from the Montreal (Quebec, Canada) area at the Douglas Mental Health University Institute from July 2021 to December 2024. All procedures were approved by the Research Ethics Board of the Centre Intégré Universitaire de Santé et de Services Sociaux de l’Ouestde-l’île-de-Montréal, and the Montreal Neurological Institute PET Working Committee. All participants provided written informed consent after full explanation of all the study procedures, in accordance with the Declaration of Helsinki and the Tri-Council Policy Statement: Ethical Conduct for Research Involving Humans (TCPS2). The capacity to provide informed consent was assessed with the MacArthur Competence Assessment Tool-Treatment [27] for FEP and CHR participants.

PET data for participants enrolled until October 2023 (49 individuals) was previously published [9]. The present extended cohort and the relationship with stress is analyzed here for the first time.

All participants completed a comprehensive medical (health and medication history) and psychiatric screening (Structured Clinical Interview for DSM-5 Axis I Disorders [SCID-5], Research Version) [28]. Participants with FEP met SCID-5 criteria for a recent (within 3 years) diagnosis of a psychotic disorder (schizophrenia, schizophreniform disorder, schizoaffective disorder, brief psychotic episode, schizophrenia spectrum, delusional disorder, or psychosis not otherwise specified). Participants in the CHR group met diagnosis criteria for prodromal risk syndrome using the Structured Interview for Prodromal Syndromes [29]. Healthy controls did not have any past or present neurological or psychiatric illnesses and had no history of psychoactive medication use, and no first-degree family members with psychotic-related disorders.

Exclusion criteria common to all cases were any concomitant or past severe medical disorder, pregnancy and/or breastfeeding, claustrophobia, metal implants that precluded an MRI scan, exceeding the radiation exposure limit (20 mSv in a 12-month period), and current DSM-5 substance use disorder (except cannabis and nicotine) or current drug use (except cannabis and nicotine). All participants completed broad-spectrum urine drug screens.

### Clinical measures

#### Acute stress

The hassles subscale of the Hassles and Uplifts Scale [30] was used to assess acute stress on the day of the PET scan. This scale consists of a list of 117 daily hassles across life domains including family relationships, financial stability, health, work, social interactions, and personal habits. Participants were instructed to rate the severity of occurring hassles as of the preceding day of the PET scan visit. This scale has demonstrated excellent internal reliability for the full scale (r ~ 0.95) in prior studies [30]. Moreover, evidence suggests that daily hassles are better related to symptom severity than life events in schizophrenia [31]. In the current sample, the hassles scale showed good internal consistency (Cronbach’s α = 0.91). The hassles severity score was not available for 1 CHR participant.

#### Chronic stress

The self-administered Trier Inventory for Chronic Stress (TICS) [32] was used to assess chronic stress. This questionnaire evaluates 57 items across 9 life domains of chronic stress (i.e., social tension, work discontent, pressure to perform, etc.) experienced over the past three months. The TICS has demonstrated excellent psychometric properties, with Cronbach’s alpha values ranging from 0.84–0.92. In the current sample, the hassles scale showed good internal consistency (Cronbach’s α = 0.95). The TICS scores were not available for 1 FEP and 3 CHR participants.

#### Depressive symptoms

The Hamilton Depression Rating Scale (HDRS) is a clinician-rated 17-item assessment that has been used extensively in clinical research and in ordinary clinical practice to measure the severity of depressive symptoms [33]. The severity of depressive symptoms as assessed by the HDRS has been associated to SV2A deficits in vivo in a sample of participants diagnosed with stress-related disorders [24]. In the current sample, the TICS showed good internal consistency (Cronbach’s α = 0.84). The HDRS score was not available for 1 CHR participant.

#### PET and MRI acquisition and analysis

PET and MRI data acquisition have been described in detail elsewhere [9] and in the Supplement (eSupplement, Methods). Briefly, all participants underwent a dynamic 90 min [^18^F]SynVest-1 PET scan using a brain-dedicated Siemens high-resolution research tomograph (HRRT). After a transmission scan, [^18^F]SynVest-1 (5.35 ± 0.98 mCi) was injected as a bolus into the antecubital vein. An MRI scan was performed on a 3-T PRISMA-Fit research dedicated scanner (Siemens). A proton density–weighted (TE = 6 s, TR = 11 ms) and a T1-weighted brain MRI scan were acquired for anatomical delineation of the regions of interest (ROIs) and co-registration with PET scans. ROIs (Anterior cingulate cortex, orbitofrontal cortex, medial prefrontal cortex, hippocampus and ventral striatum) were automatically generated using the in-house brain parcellation software ROMI [34] on each individual’s PD-weighted MRI. PD-based delineation showed suboptimal performance upon visual inspection for the amygdala in our dataset. Therefore, this region was delineated was delineated on the T1-weighted MRI using FreeSurfer v7.4.1 ([35]; http://surfer.nmr.mgh.harvard.edu/). ROIs were selected based on their relevance on synaptic alterations related to stress [36]. MRIs were co-registered to the summed PET using a normalized mutual information metric, and the transformation was applied to the ROIs delineated on the MRI. The [^18^F]SynVest-1 binding potential with respect of a non-displaceable compartment (BP_ND_) was estimated with the simplified reference tissue model [37] using a cluster of highly pure white matter [38] (eSupplement, Methods), mostly located in the centrum semiovale (hereinafter referred as centrum semiovale) as the reference region (eFigure 2 and 3), as previously published [9].

### Statistical analyses

Statistical analyses were conducted using SPSS version 29.0 (IBM), with two-sided *p*-values < 0.05 considered statistically significant. Prior to conducting analyses, the normality of clinical variables was assessed using the Shapiro-Wilk test. Variables that did not exhibit a normal distribution were transformed using the square root method to approximate normality. Group comparisons for clinical variables of interest (Hassles, TICS, and HDRS scores) were performed using Welch ANOVA, which is robust to unequal variances across groups.

For the primary hypothesis, the effect of the interaction between stress/mood scores and study group (stress/mood x group) on synaptic density was tested using separate linear mixed models for each stress construct [39]: acute stress, chronic stress and depressive symptoms. Each model included ROI as a repeated within-participant factor with a diagonal covariance structure, consistent with prior analyses [9]. Random participant effects with random intercepts were incorporated, and the dependent variable was regional synaptic density (SynVesT-1 BP_ND_). The effects of potential confounding variables (age, sex, cannabis use, nicotine use, BMI, antipsychotic dose) were tested in all models. A significant study group by stress interaction indicated differing relationships across groups. In that case, exploratory models were conducted separately for each study group to characterize the relationship between stress constructs and synaptic density within study group. Exploratory models were not corrected for multiple comparisons.

To replicate prior analyses [9], a separate linear mixed model was used to compare regional synaptic density (SynVesT-1 BP_ND_) between groups, as we did previously [9].

In all cases, effect sizes (Cohen’s F) were calculated from the univariate F statistics provided by SPSS, with thresholds defined as small (0.10), medium (0.25), and large (0.40). Study data and code are reported in the Supplementary Information – Data and Syntax.

## Results

From the 103 participants initially enrolled, a total of 78, including 21 healthy controls, 32 CHR, and 25 FEP, completed all the study procedures and had usable PET data (see Consort Diagram in Supplement). Demographic, clinical characteristics, and PET parameters of groups are presented in Table 1. There were significant group differences in depression, exposure to stressful life events, and self-perceived chronic stress scores. All subjects tested negative in urine drug screens, except for 5 healthy controls, 10 CHR, and 6 FEP who had a positive urine drug screen for *only* cannabis. Twenty-four of 57 participants in the FEP-CHR cohort were antipsychotic-free, and the rest were either receiving 250 mg or less of chlorpromazine equivalents or less than 2 months of cumulative antipsychotic exposure, or both (eSupplement eTable 1. Antipsychotic medication)

**Table 1:** Sociodemographic and clinical variables.

Variable		HC (n = 21)	CHR (n = 32)	FEP (n = 25)	Test statistic	*p* value
Age, Mean (SD), years		23.6 (3.7)	22.9 (4.4)	26.3 (4.9)	4.34^a^	**0.02**
Male/Female, n		9/12	11/21	16/9	5.08^b^	0.08
Years of education, Mean (SD)		16.9 (4.6)	15.7 (4.8)	15.1 (4.8)	0.99	0.41
BMI, Mean (SD)		25.5 (6.1)	26.1 (7.2)	26.8 (4.7)	0.23^a^	0.80
Current substance use	Non-tobacco smokers/smokers, n	20/1	20/12	18/7	7.23^b^	**0.03**
	Non-cannabis users/cannabis users, n	16/5	22/10	19/6	0.52^b^	0.77
Antipsychotic use, AP-free/AP-current, n		N/A	22/10	2/23	21.2^b^	**0.000004**
PET Parmeters, Mean (SD)	Amount injected, mCi	5.48 (0.49)	5.42 (0.40)	5.57 (0.64)	0.57^a^	0.57
	Specific activity, mCi/µmol	3881.7 (2662.0)	3672.4 (2567.0)	3505.7 (2242.9)	0.13^a^	0.88
	Mass injected, µg	0.67 (0.45)	0.70 (0.52)	0.68 (0.39)	0.02^a^	0.98
Total sqHDRS Score, Mean (SD)		1.0 (0.97)	3.4 (0.98)	2.7 (0.95)	37.2^a^	**6.49** × **10**^**−12**^
Total TICS Score, Mean (SD)		36.9 (14.6)	60.1 (19.6)	39.6 (25.4)	10.0^a^	**0.00014**
Total Hassles Score, Mean (SD)		32.4 (16.9)	42.5 (26.9)	29.4 (18.2)	2.83^a^	0.07
Duration of illness, Mean (SD), months		N/A	N/A	12.3 (12.9)	N/A	N/A

Statistical analysis was performed using - ^a^Welch Test, ^b^X^2^.

*AP* antipsychotic, *BMI* body mass index, *CHR* clinical high risk, *N/A* not applicable, *HC* healthy controls, *FEP* first episode psychosis, *sqHDRS* square root-transformed hamilton depression rating scale (HDRS), *TICS* trier inventory for chronic stress.

### Acute stress: a generalized effect on synaptic density

**Fig. 1:**
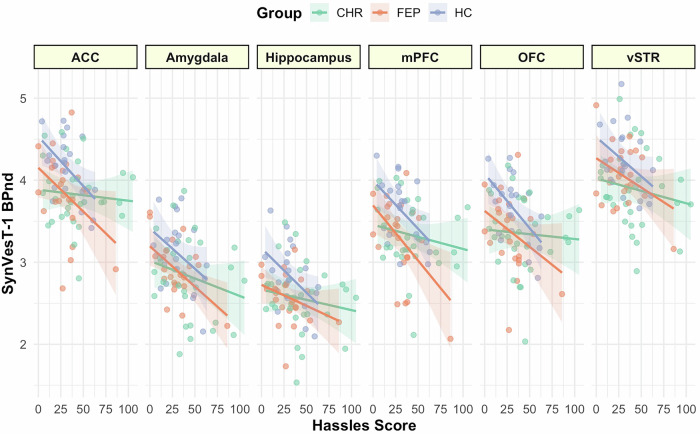
Associations between acute stress and synaptic density by study group. The panels display relationships between synaptic density as measured by [^18^F]SynVesT-1 BP_ND_ and levels of self-perceived acute stress by study group. Graphs represent raw values per ROI: ACC – Anterior cingulate cortex; Amygdala; Hippocampus; mPFC – Medial prefrontal cortex; OFC – Orbitofrontal cortex; Ventral Striatum; dots represent individual data points, lines the linear fit for each group, and shaded areas the 95% CI.

Higher self-reported acute stress was associated with lower synaptic density across all participants (Hassles Score: F_1449_ = 12.0, *p* < 0.001, Cohen’s F = 0.16; Group: F_2449_ = 3.92, *p* = 0.02; ROI: F_5449_ = 481.8, *p* < 0.001). The absence of a significant *group* × *acute stress* interaction (F_2449_ = 2.44, *p* = 0.09) suggests that the relationship between acute stress and synaptic density is not significantly different across groups (Fig. 1). These effects did not change when adjusted by potential confounding covariates (antipsychotic medication, age, sex, BMI, nicotine, and cannabis use; Table 2).

**Table 2:** Summary of covariate-adjusted models linking stress and depressive symptoms with synaptic density.

	Hassles Score^a^			Group*TICS			Group*sqHDRS		
	F	p	Cohen’s F	F	p	Cohen’s F	F	p	Cohen’s F
No covariates	12.0	**<0.01**	0.16	3.87	**0.02**	0.13	3.91	**0.02**	0.13
Antipsychotics	11.1	**<0.01**	0.16	3.92	**0.02**	0.13	3.81	**0.02**	0.13
Age	10.8	**<0.01**	0.15	3.85	**0.02**	0.13	3.75	**0.02**	0.13
Sex	12.1	**<0.01**	0.16	4.33	**0.01**	0.14	3.97	**0.02**	0.13
BMI	9.23	**<0.01**	0.15	3.09	**<0.05**	0.12	3.47	**0.03**	0.12
Nicotine	11.7	**<0.01**	0.16	3.62	**0.03**	0.13	3.55	**0.03**	0.13
Cannabis	7.76	**<0.01**	0.13	3.36	**0.04**	0.12	3.48	**0.03**	0.12

Effect of acute stress (Hassles), chronic stress (TICS) by group interaction, and depressive symptoms (squared-root transformed HDRS) by group interaction on [^18^F]SynVesT-1 BP_ND_ across all ROIs. The first row shows the unadjusted effects, subsequently the table displays changes in F, p and Cohen’s F values when adjusted by covariates. All models included diagnostic group as a covariate. ^a^Hassles scores total main effects are reported.

### Chronic stress: distinct effects across study groups

**Fig. 2:**
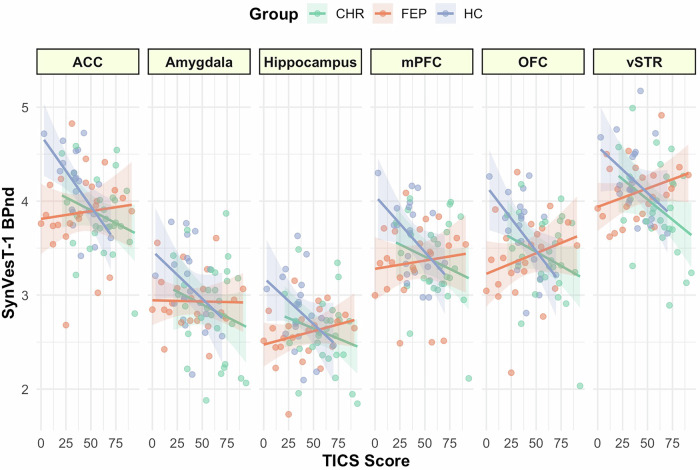
Associations between chronic stress and synaptic density by study group. The panels display relationships between synaptic density as measured by [^18^F]SynVesT-1 BP_ND_ and levels of chronic stress by study group. Graphs represent raw values per ROI: ACC Anterior cingulate cortex; Amygdala; Hippocampus; mPFC medial prefrontal cortex; OFC – Orbitofrontal cortex; Ventral Striatum; dots represent individual data points, lines the linear fit for each group, and shaded areas the 95% CI.

The relationship between chronic stress and synaptic density was different across study groups (TICS total Score*Group: F_2431_ = 3.87, *p* = 0.02; Group: F_2431_ = 5.58, *p* = 0.004; ROI: F_5431_ = 483.3, *p* < 0.001; TICS total score: F_1431_ = 4.78 *p* = 0.03). This interaction remained significant when adjusted by potential confounding covariates (antipsychotic medication, age, sex, BMI, nicotine, and cannabis use; Table 2). Exploratory within-group analyses revealed that higher perceived chronic stress was associated with lower synaptic density in healthy controls (F_1119_ = 7.07, *p* = 0.009). No significant association was observed in individuals at CHR or with FEP (CHR: F_1167_ = 2.34, *p* = 0.13, FEP: F_1135_ = 0.90, *p* = 0.35, Fig. 2). Slopes comparisons revealed significant differences between FEP and HC (eSupplement eTable 2)

### Depressive symptoms: distinct effects across study groups

**Fig. 3:**
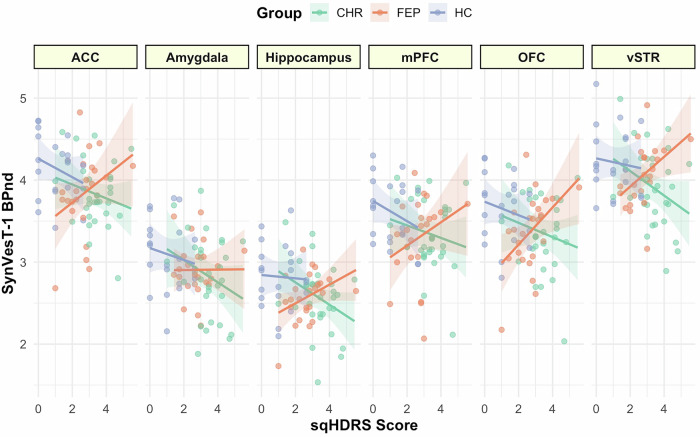
Associations between severity of depressive symptoms and synaptic density by study group. The panels display relationships between synaptic density as measured by [^18^F]SynVesT-1 BP_ND_ and severity of depressive symptoms by study group. Graphs represent raw values per ROI: ACC – Anterior cingulate cortex; Amygdala; Hippocampus; mPFC – Medial prefrontal cortex; OFC Orbitofrontal cortex; Ventral Striatum; dots represent individual data points, lines the linear fit for each group, and shaded areas the 95% CI.

We observed a significant interaction between study group and depressive symptoms (sqHDRS total Score*Group: F_2449_ = 3.91, *p* = 0.02; Group: F_2449_ = 5.55, *p* = 0.004; ROI: F_5449_ = 479.35, *p* < 0.001; sqHDRS total score: F_1449_ = 0.001 *p* = 0.97). This interaction remained significant when adjusted by potential confounding covariates (antipsychotic medication, age, sex, BMI, nicotine, and cannabis use; Table 2). Exploratory within-group analyses revealed that higher depressive symptoms were associated with higher synaptic density in FEP (F_(1141)_ = 5.19, *p* = 0.02). No significant association was observed in individuals at CHR or in healthy controls (CHR: F_(1179)_ = 1.63, *p* = 0.20, HC: F_(1119)_ = 0.99, *p* = 0.32, Fig. 3). Slopes comparisons revealed significant differences between FEP and HC (eSupplement eTable 3)

## Discussion

To our knowledge, this is the first study to evaluate the impact of stress on synaptic density in psychotic-related disorders. We found that acute stress severity was significantly associated with lower synaptic density across all study groups. In contrast, and contrary to our hypotheses, chronic stress was linked to synaptic density deficits only in healthy controls, whereas no such effect was observed in individuals with CHR or FEP. Paradoxically, we observed that greater depressive symptomatology was associated with higher synaptic density in FEP patients, with no effect in CHR or healthy controls. Finally, consistent with our prior findings, we confirmed a significant effect of group on synaptic density in this larger cohort, showing lower synaptic density in clinical groups (eResult 1).

Previous in vivo studies have provided evidence of a negative relationship between synaptic density and stress severity, so far in individuals without psychotic disorders. For example, Holmes et al. reported lower synaptic density ([¹¹C]UCB-J volume of distribution (V_T_)) in the dorsolateral prefrontal cortex, hippocampus, and anterior cingulate cortex in patients diagnosed with stress-related disorders and undergoing severe depression, compared to healthy controls [24]. In a post-traumatic stress disorder (PTSD) subgroup, depressive symptoms were negatively correlated with synaptic density in these same regions [24]. Moreover, Asch et al reported a negative correlation between negative affect and synaptic density [¹¹C]UCB-J V_T_ in patients diagnosed with mood and stress-related disorders ( ~ 50% also meeting obesity criteria) which was also associated with lower synaptic density [40]. Observations from animal models have also shown reductions in synaptic density ([^18^F]SynVesT-1 BP_ND_) in the prefrontal cortex and hippocampus following chronic stress exposure in male and female rats [23].

Taken together, these prior studies suggest that synaptic density plays a key role in stress processing and emotional regulation in non-psychotic populations. Our findings extend this line of research by showing that, in psychotic-related disorders the relationship between stress/mood and synaptic integrity is disrupted.

Whereas prior work suggests that greater depressive symptomatology is associated with synaptic density deficits [24]; we observed that in FEP patients, higher depressive symptoms were associated with greater synaptic density. While this is, to our knowledge, the first in vivo PET study to report such relationship, some prior evidence points in a similar direction. For example, postmortem studies showed that while patients with schizophrenia had lower pre-synaptic proteins [41] compared to healthy controls; schizophrenia patients *who died by suicide* showed higher immunoreactivity for pre-synaptic proteins than those who died from other causes in the anterior prefrontal region [41, 42]. In line with postmortem studies, a structural MRI study reported *greater* cortical thickness in CHR individuals with suicide ideation compared to those without [43]. In schizophrenia, despite the broader literature on cortical thinning [44], only two studies have specifically compared individuals with and without suicide attempts [45, 46], and showed differing results. Besteher et al., reported *lower* cortical thickness in 14 patients with schizophrenia and history of suicide attempts compared to non-attempters in several brain regions [45]. A subsequent larger study (n = 37 schizophrenia suicide attempters, n = 37 non-attempters) found significant *greater* cortical thinning only in temporal regions, with no differences in frontal areas [46]. Here, we observed that more depressive symptoms were associated with greater synaptic density in medial frontal regions, consistent with postmortem studies [4, 5], but partially inconsistent with cortical thickness reports [43, 45, 46]. Notably, cortical thickness is influenced by other factors beyond synaptic density [47], and meta-analyses of postmortem studies have yielded inconclusive results regarding synaptic density alterations in temporal regions [4, 5].

Overall, these findings suggest that the neurobiological mechanisms supporting adaptation to prolonged stress are altered early in the course of psychotic illnesses, either as a consequence of disease-related changes or as part of an underlying vulnerability. Other lines of evidence also suggest that stress response patterns differ across disorders. For instance, individuals with non-affective psychosis show heightened vulnerability to daily hassles, exhibiting increased negative affect and reduced positive affect [48]. Conversely, depressed patients display increases in negative affect only [48]. Furthermore, early stages of psychosis have been consistently linked to a blunted cortisol awakening response [49], whereas individuals with depression often show an exaggerated response [50]. We initially hypothesized no association in healthy controls, given their relative resilience to stress. However, the observed relationship may reflect a normative, adaptive neurobiological response—one that is disrupted in the context of psychotic illness.

A metanalysis of studies that reported on hypothalamic–pituitary–adrenal (HPA) axis reactivity to psychological stressors revealed differences in the activation pattern of individuals with schizophrenia compared to affective disorders [51]. While depressed patients display cortisol responses to acute stress comparable to those of healthy controls, in schizophrenia the cortisol increase was less pronounced [51]. Although we may speculate that psychotic states would alter the subjective experience to stress, studies show that patients with psychosis rate stress-inducing tasks as upsetting as controls [52] and exhibit similar autonomic responses [53].

Alternatively, our findings may reflect the neurobiological heterogeneity within psychotic disorders. Prior work suggests that lower grey matter density is linked to psychosis subgroups characterized by greater cognitive deficits [54, 55]. Since we also demonstrated lower synaptic density in those with higher negative symptoms’ severity [9], these observations raise the possibility that lower synaptic density may index a subgroup of individuals with deficit-related symptoms.

In our study, we did not find significant associations between chronic stress and synaptic density deficits in individuals with psychosis. This finding challenges our previous assumption that stress depletes synaptic integrity. One possible explanation lies in altered HPA axis functioning. While chronic elevations in cortisol have been linked to synaptic loss [56], animal studies also show that finely tuned glucocorticoid oscillations are essential for learning-dependent synapse formation and maintenance [57]. Disruptions in this regulatory balance may therefore lead to atypical synaptic responses to stress in psychosis, potentially reflecting compensatory or maladaptive neuroplasticity.

This study shares several methodological limitations previously discussed in Blasco et al. [9], given the use of similar acquisition and analysis procedures. Specifically, while SV2A is a well-established marker of synaptic density due to its strong correlation with synaptophysin, it may also reflect changes in synaptic vesicle production, SV2A protein structure, or vesicular membrane composition [11]. Second, we used a simplified reference tissue model (SRTM) [37] with a pure white matter region as the reference region [58]. Prior studies demonstrated a near-perfect linear relationship across brain regions (R² = 1.00) between [^18^F]SynVesT-1 BP_ND_ estimated from white matter V_T_ and BP_ND_ estimated with V_ND_ obtained via Lassen occupancy plots, supporting its use [59]. However, binding potential could be underestimated differently between groups, if the V_T_ of the white matter is different across groups. Consistent with our previous findings [9], we found no differences in white matter time-activity curves across our larger study groups (eSupplementary Fig. 2). Here, we also report no differences when comparing depressed vs non-depressed participants (eSupplementary eFig. 3). Previous studies have also reported no differences in the white matter [^11^C]UCB-J V_T_ when comparing patients with psychotic disorders and healthy controls [7, 8].

Third, stress was primarily assessed using self-report measures (e.g., Hassles Scale and TICS), and no other physiological indicators (such as cortisol or responses to standardized stress tasks) are available. While one could speculate that psychotic states imply reduced reliability in self-reporting stress, we also observed similar associations with mood symptoms as assessed by the clinician-rated HDRS; and previously self-reported stress levels were consistent with clinician-rated measures (HDRS) [60]. These ecological and subjective tools may have particular clinical relevance, as they reflect what is commonly available in real-world psychiatric settings. Our findings suggest that such accessible stress measures may be meaningfully linked to underlying molecular markers.

Finally, due to the observational and cross-sectional design, causality between stress/mood and synaptic density cannot be determined. We do not know whether pre-existing higher synaptic density is a risk factor for the subsequent development of mood or stress related disorders in those with emerging psychosis or FEP, or rather a consequence of these experiences. Longitudinal studies are needed to examine whether sustained stress exposure in the prodromal phase impacts synaptic density longitudinally, potentially contributing to disrupted stress-synapse associations in those who develop full psychosis or schizophrenia. Alternatively, early synaptic dysfunction—particularly in emotion regulation circuits—could lead to altered stress perception, especially in those at risk for psychosis. In addition, although we carefully evaluated the influence of relevant covariates, the inclusion of all potential confounders simultaneously in a single model was not possible due risk of overfitting and/or unstable estimates.

In summary, our findings suggest that the relationship between stress and synaptic density is altered in psychotic disorders, potentially reflecting impaired adaptive neuroplasticity. Future studies should confirm our findings in independent samples. Moreover, longitudinal studies are needed to clarify causal pathways and support the development of targeted interventions to mitigate stress-related synaptic dysfunction.

## Supplementary information

Supplementary Information

Supplementary Information – Data and Syntaxis

## Data Availability

Study data and code are reported in the Supplementary Information – Data and Syntax.
