# Supplementary material for: Stress and synaptic density in psychosis and clinical high risk: evidence from [18F]SynVesT-1 PET
Source: Transl Psychiatry. 2026 Apr 15;16:277. doi: 10.1038/s41398-026-03993-9 (PMC13194929; doi:10.1038/s41398-026-03993-9)
Supplement: Supplementary file 1 — Supplementary Information [file 41398_2026_3993_MOESM1_ESM.docx]

**Supplementary Information**

**eMethods**

**eFigure 1.** Schematic participant’s Consort Diagram

**eTable 1.** Antipsychotic medication description

**eTable 2**. Slopes of [^18^F]SynVesT-1 BP_ND_ vs. TICS score comparison by study group

**eTable 3**. Slopes of [^18^F]SynVesT-1 BP_ND_ vs. sqHDRS score comparison by study group

**eFigure 2.** [^18^F]SynVesT-1 time activity curves expressed in standard uptake values (SUV) in white matter between groups

**eFigure 3.** [^18^F]SynVesT-1 time activity curves expressed in standard uptake values (SUV) in white matter between depressed and non-depressed individuals.

**eFigure 4.** Synaptic density [^18^F]SynVesT-1 in First Episode Psychosis (FEP) patients and Clinical High-Risk individuals across brain regions (ROIs).

**eResult 1.** Group effect on synaptic density ([^18^F]SynVesT-1 BP_ND_)

**eResult 2.** Sensitivity analyses excluding participants for whom only 80 minutes of PET data were used (n=3).

eSupplement

Methods

PET and MRI image acquisition

Each participant underwent a dynamic 90-minute [^18^F]SynVest-1 PET scan using a Siemens high-resolution research tomograph (HRRT), which acquires 207 slices with a 1.2-mm separation. To minimize head movement, a Velcro strap was utilized in conjunction with a head fixation system. Prior to tracer administration, a single photon point source ^137^Cs transmission scan was performed for attenuation correction.

After the transmission scan, [^18^F]SynVest-1 (198.08 ± 36.05 MBq) was injected as a bolus into the antecubital vein. Images were reconstructed into a series of 28-time frames defined as 4x10 sec, 4x20 sec, 3x40 sec, 3x60 sec, 4x120 sec, 5x300 sec, 5x600 sec. Image reconstruction was performed using the ordered-subsets expectation maximization (OSEM) algorithm with a three dimensional approach (OSEM3D-OP-PSF), 16 subsets and 10 iterations^1^ with a 207x256x256 matrix and isotropic voxels of 1.22 mm^3^. OSEM accounts for detector channel normalization, attenuation, random coincidences, scattered radiation, dead time and decay corrections. The reconstruction algorithm used accounted for motion and point spread function, therefore correcting for partial volume effects. Following the method validated by Costes *et al*^2^, the images were reconstructed twice to correct for potential transmission and emission scan misalignment. Additionally, all the images underwent a quality control before analysis. In three cases, the motion in specific final frames was too large and produced a systematic bias in the regional time activity curves (TACs). For these subjects, only 80 min of data was used in the SRTM fitting. Another participant, that excited the scanner twice, was not considered for final analysis due to excessive motion and missing data points in the TAC (see Consort diagram, eFigure 1 and eResult 2).

A structural magnetic resonance imaging (MRI) scan was conducted using a 3T Siemens PRISMA-Fit research-dedicated scanner at the Douglas Cerebral Imaging Center (CIC). A proton density (PD)-weighted brain MRI scan was acquired for anatomical delineation of the regions of interest (ROIs) and co-registration with PET scans. All images were acquired with the following parameters: repetition time = 6000 ms, echo time = 11 ms, flip angle = 125°, 86 slices of 1.5 mm thickness, distance factor = 30%, voxel size = 0.3 × 0.3 × 1.5 mm.

**PET Imaging Analysis**

PET Image analysis was performed with an in-house software ROMI^3^. The software uses a standard brain template (International Consortium for Brain Mapping/Montreal Neurological Institute 152 MRI) containing a set of predefined cortical and subcortical ROIs (based upon the neuroanatomy atlas of Duvernoy^4^ but consistent with the atlas of Talairach^5^; the division of the striatum is from Mawlawi et al.^6^ and the prefrontal cortex subregions are derived from the cytoarchitectural definitions of Rajkowska)^7–9^. The amygdala was delineated using FreeSurfer^10^ (v7.4.1; <http://surfer.nmr.mgh.harvard.edu/>), employing the standard recon-all processing pipeline applied to T1-weighted structural MRI scans. This pipeline performs a fully automated processing including motion correction, skull stripping, intensity normalization, Talairach transformation, segmentation of subcortical structures, cortical surface reconstruction, and anatomical parcellation. Each subject's MRI was co-registered to their summed [^18^F]SynVesT-1 PET images with a rigid body transformation using the normalized mutual information algorithm. The transformations were then applied to the ROIs that were delineated in the MRI native space. The resliced ROIs were used to mask the [^18^F]SynVesT-1 PET image and generate the TAC. The simplified reference tissue model^11^ was employed with the 90-minute TAC to estimate the BP_ND_, using a highly pure with matter mostly corresponding to the centrum semiovale (see below) as the reference region to compute regional BP_ND_.

**White matter as reference region (eFigure 2)**

White matter has been proposed as a suitable reference region due to its negligible presence of SV2A in the baboon brain^12^. The centrum semiovale is a region characterized for the presence of mostly white matter and has been used as a reference region (V_T_) to measure SV2A BP_ND_ in gray matter regions using both [^11^C]UCB-J^12^ and [^18^F]SynVesT-1^13^. Even though low percentages of displaceable binding were previously found in this white matter region (about 10% of the V_T_^13^), it was demonstrated a constant relationship between centrum semiovale V_T_ and grey matter V_ND_^13^. Therefore, it was proposed that, assuming that white matter is not affected by the studied condition, the centrum semiovale can serve as a reference region^14^.

In our study, white matter region was delineated as described by Bencherif et al^15^. In summary, each subject-specific MR image was segmented probabilistically into three compartments, grey matter, white matter, and cerebrospinal fluid using SPM2. An MRI native resolution white matter region was created by keeping only voxels with high probability (*p*>90). The MRI was co-registered to the summed PET image. The transformation was used to create the WM region in the PET space. The new ROI was smoothed using a 3-dimensional gaussian kernel with FWHM=5 mm (which is higher than the resolution of the scanner with the described reconstruction algorithm). Then, the image was resliced by a nearest-neighbor interpolation. Another threshold (*p*>0.95) was applied to get a cluster of WM less affected by partial volume effects. In the practice this cluster usually corresponds to the centrum semiovale.

Previously published studies demonstrated no significant differences in V_T_ in the centrum semiovale between samples with schizophrenia or first episode psychosis and healthy controls^16,17^. Here, we compared the TAC standardized uptake values (SUV) measured in the centrum semiovale between our study groups (eFigure 2). We designed a linear mix model with study group (HV, CHR, and FEP) as fixed factor; time (28 different time-point measurements) as a repeated within-subject fixed factor with a diagonal covariance structure; participants as random effects, and SUV as the dependent variable. [^18^F]SynVesT-1 uptake did not significantly differ between groups (F_(2,1116)_=0.631, *p*=0.53), supporting the use of our reference.

**eFigure 1 – Consort diagram.** The flow diagram describes reasons for outcome data exclusion by study group.


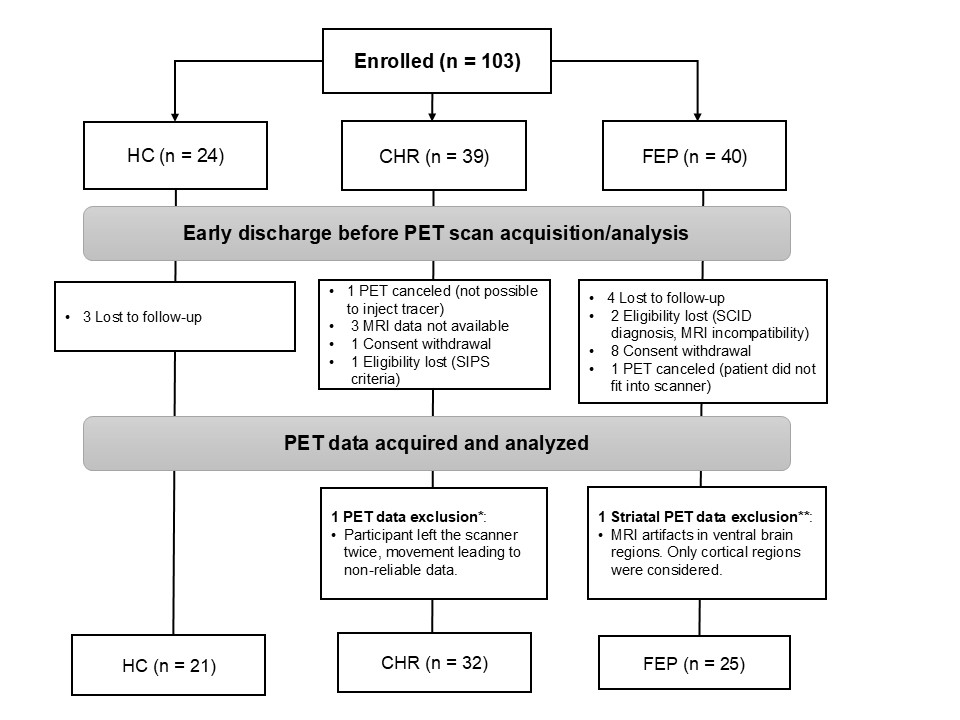


Abbreviations: CHR – Clinical High Risk; HC – Health Controls; FEP – First-episode Psychosis; N – sample size; PET - positron emission tomography.

* The participant exited the PET scanner at 34 minutes (for 3 minutes) and later at 65 minutes (for 5 minutes) making SV2A BP_ND_ estimations unreliable.

** The participant had dental braces, hence only cortical regions were considered for BP_ND_ measurements

**eTable 1. Antipsychotic medication.** The table describes characteristics of antipsychotic medication for each participant.

|  | **Subject** | **Current Antipsychotic** | **Daily dose (mg)** | **Chlorpromazine equivalent dose (mg)** |
| --- | --- | --- | --- | --- |
| **CHR** | 1 | Naive | n/a | n/a |
|  | 2 | None | n/a | n/a |
|  | 3 | Quetiapine | 50 | 29.4 |
|  | 4 | Naive | n/a | n/a |
|  | 5 | Naive | n/a | n/a |
|  | 6 | Lurasidone | 40 | 250 |
|  | 7 | Naive | n/a | n/a |
|  | 8 | Quetiapine | 25 | 13.0 |
|  | 9 | Naive | n/a | n/a |
|  | 10 | Quetiapine | 25 | 13.0 |
|  | 11 | Naive | n/a | n/a |
|  | 12 | Naive | n/a | n/a |
|  | 13 | Quetiapine | 25 | 13.0 |
|  | 14 | Olanzapine | 2.5 | 48.1 |
|  | 15 | Naive | n/a | n/a |
|  | 16 | Quetiapine | 100 | 66.3 |
|  | 17 | Risperidone | 2 | 154.6 |
|  | 50 | Naive | n/a | n/a |
|  | 52 | Naive | n/a | n/a |
|  | 53 | Naïve | n/a | n/a |
|  | 55 | Naïve | n/a | n/a |
|  | 56 | Naïve | n/a | n/a |
|  | 59 | Naive | n/a | n/a |
|  | 65 | Naive | n/a | n/a |
|  | 66 | None | n/a | n/a |
|  | 67 | Quetiapine | 25 | 13.0 |
|  | 69 | Naive | n/a | n/a |
|  | 71 | Lurasidone  Olanzapine | 40  10 | 486.7* |
|  | 72 | Naive | n/a | n/a |
|  | 74 | None | n/a | n/a |
|  | 75 | Olanzapine  Aripiprazole | 5  10 | 295.3* |
|  | 77 | Aripiprazole | 2 | 19.0 |
| **FEP** | 18 | Olanzapine | 5 | 106.7 |
|  | 19 | Aripiprazole | 5 | 70.2 |
|  | 20 | Aripiprazole | 2.5 | 26.1 |
|  | 21 | Lurasidone | 20 | 125 |
|  | 22 | Aripiprazole | 5 | 70.2 |
|  | 23 | Lurasidone | 40 | 250 |
|  | 24 | Aripiprazole | 7 | 113.5 |
|  |  | Quetiapine | 25 | 13.0 |
|  | 25 | None | n/a | n/a |
|  | 26 | Quetiapine | 25 | 13 |
|  | 27 | Risperidone | 3 | 239.4 |
|  | 28 | Aripiprazole | 10 | 237.0 |
|  |  | Olanzapine | 2.5 |  |
|  | 29 | Paliperidone | 5.2 | 326* |
|  | 30 | Risperidone | 1.5 | 113 |
|  | 31 | Aripiprazole | 10 | 188.9 |
|  | 32 | Olanzapine | 5 | 106.7 |
|  | 33 | Olanzapine  Quetiapine | 10  150 | 342.0* |
|  | 51 | Olanzapine | 5 | 106.7 |
|  | 54 | Aripiprazole | 5 | 70.2 |
|  | 58 | Olanzapine | 5 | 106.7 |
|  | 60 | Naïve | n/a | n/a |
|  | 61 | Aripiprazole | 20 | 280.8* |
|  | 62 | Paliperidone  Risperidone  Quetiapine | 5.2  1  75 | 609.2* |
|  | 73 | Aripiprazole | 2 | 19.0 |
|  | 76 | Quetiapine | 50 | 29.4 |
|  | 78 | Brexiprazole | 2 | 200.0 |
|  | mean ± SD dose | |  | **145.9 ± 144.6** |
| Medication received at the time of the PET scan is described. *Medication received < 2 months   \| **eTable 2**. **Slopes of [^18^F]SynVesT-1 BP_ND_ vs. TICS score comparison by study group** \| \| \| \| \| --- \| --- \| --- \| --- \| \|  \| Slope difference (β) \| *p* value \| IC 95 % \| \| FEP vs HC \| .016 \| 0.01 \| .004, .028 \| \| CHR vs HC \| .007 \| 0.26 \| -.006, .020 \|   **eTable 3**. **Slopes of [^18^F]SynVesT-1 BP_ND_ vs. sqHDRS score comparison by study group**   \|  \| Slope difference (β) \| *p* value \| IC 95 % \| \| --- \| --- \| --- \| --- \| \| FEP vs HC \| .269 \| 0.02 \| .036, .502 \| \| CHR vs HC \| -.007 \| 0.95 \| -.227, .213 \| | | | | |

| **eFigure 2. [^18^F]SynVesT-1 time activity curves expressed in standard uptake values (SUV) in white matter between groups.** [^18^F]SynVesT-1 uptake did not significantly differ between groups (F_(2,2151)_ = 1.01, p = 0.36).   |
| --- |

| **eFigure 3. [^18^F]SynVesT-1 time activity curves expressed in standard uptake values (SUV) in white matter between depressed and non-depressed individuals.** White matter [^18^F]SynVesT-1 uptake in individuals reaching HDRS (Hamilton Depression Rating Scale) depression threshold (>= 7 HDRS total score) was not significantly different compared to individuals showing negligible depressive symptoms (<7 HDRS total score; F_(1,2123)_ = 0.006, p = 0.94).    **eFigure 4. Synaptic density [^18^F]SynVesT-1 in First Episode Psychosis (FEP) patients and Clinical High-Risk individuals across brain regions (ROIs).** [ 18F]SynVesT-1 binding potential values represent raw data, and horizontal bars indicate the group mean.  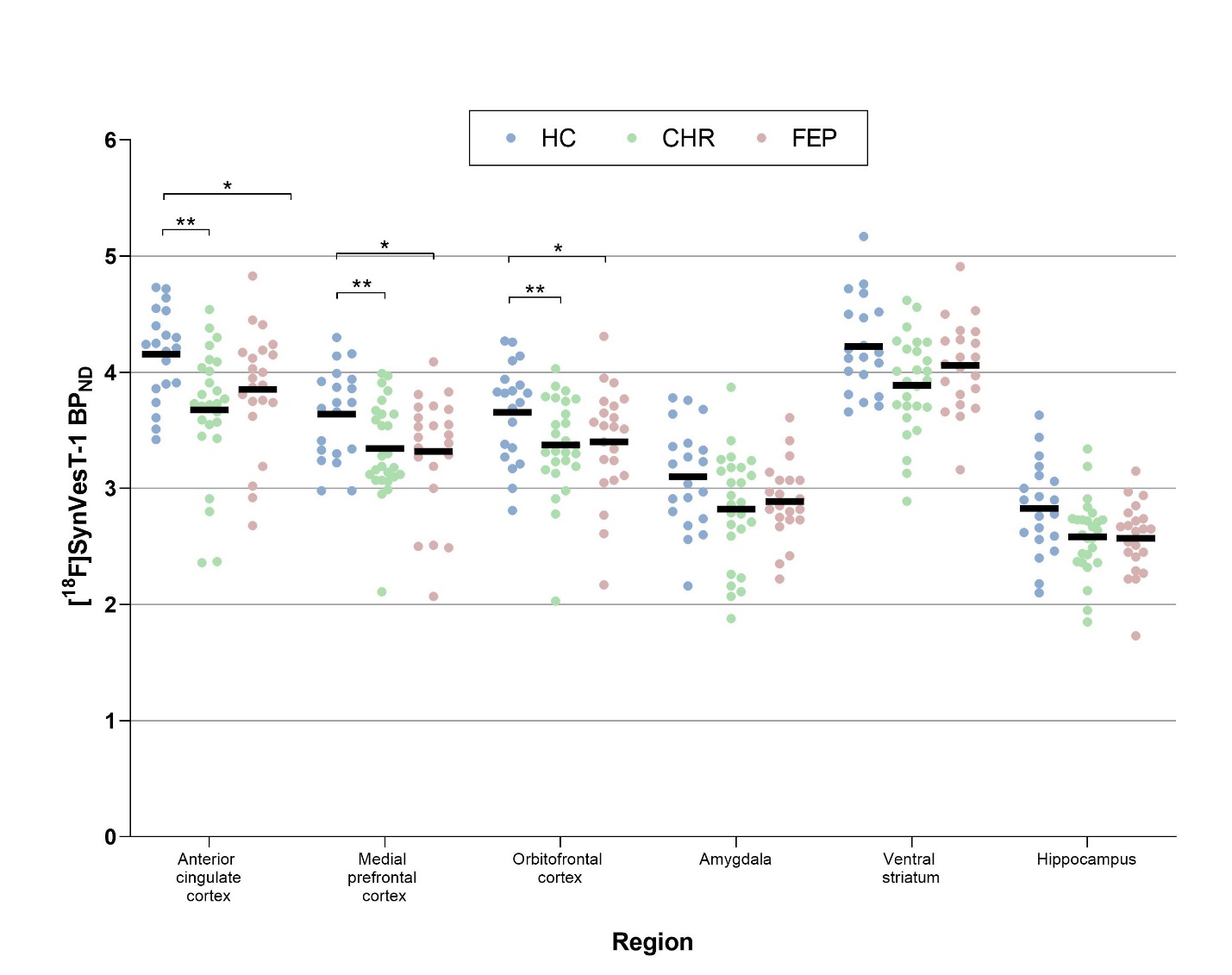  Abbreviations: CHR – Clinical High-Risk , HC – Healthy Controls, FEP – First Episode Psychosis. *Indicates statistical significance at FDR-adjusted *p* < 0.05 in regions comparing FEP vs HC (Mean Difference [SE], p value – Anterior cingulate cortex: -0.30 [0.12], 0.02; Medial prefrontal cortex: -0.32 [0.12], 0.02; Orbitofrontal cortex: -0.28 [0.12], 0.03). **Indicates statistical significance at FDR-adjusted *p* < 0.05 in regions comparing CHR vs HC (Mean Difference [SE], p value – Anterior cingulate cortex: -0.31 [0.11], 0.02; Medial prefrontal cortex: -0.27 [0.12], 0.04; Orbitofrontal cortex: -0.29 [0.11], 0.04). |
| --- |

**eResult 1. Group effect on synaptic density ([^18^F]SynVesT-1 BP_ND_)**

Consistent with our previous findings, we also observed a significant effect of group on synaptic density in this larger cohort (Group: F_(2,458)_= 3.99, p = 0.02, Cohen’s F = 0.13; ROI: F_(5,450)_= 491.3 p < .001, Cohen’s F = 1.48). However, here the interaction group by ROI was tested and found non-significant (F_(2,448)_= 0.49, p = 0.89), suggesting that the effect of group is similar across all the ROIs considered for this analysis. Post-hoc pairwise comparisons revealed significant lower synaptic density in anterior cingulate cortex, medial prefrontal cortex, and orbitofrontal cortex for both CHR and FEP individuals compared to healthy controls (eFigure 3).  Group effects remained significant when adjusted by these covariates (data now shown).

**eResult 2. Sensitivity analyses excluding participants for whom only 80 minutes of PET data were used (n=3).**

**2.1 Acute stress (Hassles)**

Higher acute stress remained significantly associated with lower synaptic density across all participants (Hassles Score: F_(1,431)_ = 12.2, *p* < 0.001; Group: F_(2,431)_ = 3.53, *p* = 0.03; ROI: F_(5,431)_ = 462.2, *p* < 0.001). Consistently, the group × acute stress interaction (F_(2,431)_ = 2.17, *p* = 0.11) was non-significant.

**2.2 Chronic stress (TICS)**

The relationship between chronic stress and synaptic density also continued to significantly differ across study groups (TICS total score × group: F_(2,413)_ = 3.81, *p* = 0.023; Group: F_(2,413)_ = 5.54, *p* = 0.004; ROI F_(5,413)_ = 462.86, *p* < 0.001; TICS total score: F_(1,413)_ = 4.71, *p* = 0.03).

**2.3 Depressive symptoms (HDRS)**

A significant interaction between depressive symptoms and study group was also preserved (sqHDRS × group: F_(1,431)_ = 4.14, *p* = 0.02; Group: F_(1,431)_ = 5.62, *p* = 0.004; ROI: F_(5,431)_ = 460.00, *p* < 0.001; sqHDRS: F_(1,431)_= 0.03, *p* = 0.86).

**References**

1. Hong IK, Chung ST, Kim HK, Kim YB, Son YD, Cho ZH. Ultra Fast Symmetry and SIMD-Based Projection-Backprojection (SSP) Algorithm for 3-D PET Image Reconstruction. *IEEE Trans Med Imaging*. 2007;26(6):789-803. doi:10.1109/TMI.2007.892644

2. Costes N, Dagher A, Larcher K, Evans AC, Collins DL, Reilhac A. Motion correction of multi-frame PET data in neuroreceptor mapping: Simulation based validation. *NeuroImage*. 2009;47(4):1496-1505. doi:10.1016/j.neuroimage.2009.05.052

3. Rusjan P, Mamo D, Ginovart N, et al. An automated method for the extraction of regional data from PET images. *Psychiatry Res Neuroimaging*. 2006;147(1):79-89. doi:10.1016/j.pscychresns.2006.01.011

4. Duvernoy HM. *The Human Brain*. Springer Vienna; 1999. doi:10.1007/978-3-7091-6792-2

5. Talairach J, Tournoux P, Rayport M. *Co-Planar Stereotaxic Atlas of the Human Brain: 3-Dimensional Proportional System: An Approach to Cerebral Imaging*. Reprint. Thieme [u.a.]; 1997.

6. Mawlawi O, Martinez D, Slifstein M, et al. Imaging Human Mesolimbic Dopamine Transmission with Positron Emission Tomography: I. Accuracy and Precision of D _2_ Receptor Parameter Measurements in Ventral Striatum. *J Cereb Blood Flow Metab*. 2001;21(9):1034-1057. doi:10.1097/00004647-200109000-00002

7. Rajkowska G, Goldman-Rakic PS. Cytoarchitectonic Definition of Prefrontal Areas in the Normal Human Cortex: I. Remapping of Areas 9 and 46 using Quantitative Criteria. *Cereb Cortex*. 1995;5(4):307-322. doi:10.1093/cercor/5.4.307

8. Rajkowska G, Goldman-Rakic PS. Cytoarchitectonic Definition of Prefrontal Areas in the Normal Human Cortex: II. Variability in Locations of Areas 9 and 46 and Relationship to the Talairach Coordinate System. *Cereb Cortex*. 1995;5(4):323-337. doi:10.1093/cercor/5.4.323

9. Uylings HBM, -Arigita EJS, De Vos K, Pool CW, Evers P, Rajkowska G. 3-D Cytoarchitectonic parcellation of human orbitofrontal cortex. *Psychiatry Res Neuroimaging*. 2010;183(1):1-20. doi:10.1016/j.pscychresns.2010.04.012

10. Fischl B. FreeSurfer. *NeuroImage*. 2012;62(2):774-781. doi:10.1016/j.neuroimage.2012.01.021

11. Lammertsma AA, Hume SP. Simplified Reference Tissue Model for PET Receptor Studies. *NeuroImage*. 1996;4(3):153-158. doi:10.1006/nimg.1996.0066

12. Finnema SJ, Nabulsi NB, Eid T, et al. Imaging synaptic density in the living human brain. *Sci Transl Med*. 2016;8(348). doi:10.1126/scitranslmed.aaf6667

13. Naganawa M, Li S, Nabulsi N, et al. First-in-Human Evaluation of ^18^ F-SynVesT-1, a Radioligand for PET Imaging of Synaptic Vesicle Glycoprotein 2A. *J Nucl Med*. 2021;62(4):561-567. doi:10.2967/jnumed.120.249144

14. Rossano S, Toyonaga T, Finnema SJ, et al. Assessment of a white matter reference region for ^11^ C-UCB-J PET quantification. *J Cereb Blood Flow Metab*. 2020;40(9):1890-1901. doi:10.1177/0271678X19879230

15. Bencherif B, Stumpf MJ, Links JM, Frost JJ. Application of MRI-based partial-volume correction to the analysis of PET images of mu-opioid receptors using statistical parametric mapping. *J Nucl Med Off Publ Soc Nucl Med*. 2004;45(3):402-408.

16. Onwordi EC, Whitehurst T, Shatalina E, et al. Synaptic Terminal Density Early in the Course of Schizophrenia: An In Vivo UCB-J Positron Emission Tomographic Imaging Study of Synaptic Vesicle Glycoprotein 2A. *Biol Psychiatry*. Published online June 2023:S0006322323013537. doi:10.1016/j.biopsych.2023.05.022

17. Onwordi EC, Halff EF, Whitehurst T, et al. Synaptic density marker SV2A is reduced in schizophrenia patients and unaffected by antipsychotics in rats. *Nat Commun*. 2020;11(1):246. doi:10.1038/s41467-019-14122-0
