# Supplementary material for: Stress and synaptic density in psychosis and clinical high risk: evidence from [18F]SynVesT-1 PET
Source: Transl Psychiatry. 2026 Apr 15;16:277. doi: 10.1038/s41398-026-03993-9 (PMC13194929; doi:10.1038/s41398-026-03993-9)
Supplement: Supplementary file 2 — Supplementary Information – Data and Syntaxis [file 41398_2026_3993_MOESM2_ESM.docx]

**Supplementary Information – Data and Syntaxis**

| ID | Group | HDRS | Hassless | TICS | ACC_SV2A | HIPP_SV2A | mPFC_SV2A | OFC_SV2A | Amyg_SV2A | vSTR_SV2A |
| --- | --- | --- | --- | --- | --- | --- | --- | --- | --- | --- |
| 1 | CHR | 11 | 7 | 76 | 4.54 | 2.74 | 3.99 | 3.84 | 3.41 | 4.18 |
| 2 | CHR | 23 | 93 | 89 | 3.57 | 1.95 | 2.95 | 3.24 | 2.11 | 3.13 |
| 3 | CHR | 18 | 5 | 36 | 3.84 | 2.43 | 3.64 | 3.41 | 2.59 | 4.10 |
| 4 | CHR | 7 | 5 | 33 | 4.30 | 2.71 | 3.91 | 3.88 | 2.88 | 4.02 |
| 5 | CHR | 12 | 36 | 55 | 3.73 | 2.57 | 3.16 | 3.23 | 2.86 | 3.88 |
| 6 | CHR | 30 | 31 | 75 | 4.38 | 2.79 | 3.97 | 4.03 | 3.25 | 4.20 |
| 7 | CHR | 8 | 33 | 54 | 3.66 | 2.49 | 3.14 | 3.16 | 1.88 | 3.71 |
| 8 | CHR | 9 | 11 | 63 | 3.45 | 2.73 | 3.06 | 3.31 | 3.05 | 3.79 |
| 9 | CHR | 9 | 38 | 74 | 4.01 | 3.34 | 3.76 | 3.79 | 3.87 | 4.56 |
| 10 | CHR | NA | 94 | NA | 4.09 | 2.67 | 3.54 | 3.56 | 3.18 | 4.01 |
| 11 | CHR | 18 | 105 | 72 | 4.04 | 2.57 | 3.67 | 3.64 | 2.78 | 3.71 |
| 12 | CHR | 9 | 19 | 75 | 3.77 | 3.19 | 3.18 | 3.32 | 3.15 | 3.70 |
| 13 | CHR | 22 | 45 | 94 | 2.80 | 1.85 | 2.11 | 2.03 | 2.07 | 3.24 |
| 14 | CHR | 7 | NA | 86 | 4.11 | 2.72 | 3.84 | 3.77 | 3.18 | 4.27 |
| 15 | CHR | 17 | 45 | 72 | 3.43 | 2.12 | 3.19 | 2.98 | 2.23 | 2.89 |
| 16 | CHR | 11 | 70 | 69 | 3.73 | 2.84 | 3.12 | 3.55 | 2.16 | 4.26 |
| 17 | CHR | 9 | 26 | 32 | 3.55 | 2.73 | 3.10 | 3.31 | 3.11 | 4.26 |
| 18 | FEP | 7 | 31 | 45 | 4.19 | 2.41 | 3.70 | 3.54 | 2.73 | 3.86 |
| 19 | FEP | 12 | 28 | 38 | 3.81 | 2.74 | 3.29 | 3.25 | 2.73 | 4.35 |
| 20 | FEP | 9 | 0 | 64 | 4.41 | 2.97 | 3.83 | 3.95 | 3.61 | 4.91 |
| 21 | FEP | 3 | 16 | 20 | 4.24 | 2.54 | 3.68 | 3.61 | 2.91 | 4.13 |
| 22 | FEP | 3 | 23 | 17 | 3.75 | 2.45 | 3.35 | 3.40 | 2.82 | 3.66 |
| 23 | FEP | 11 | 27 | 74 | 4.12 | 2.72 | 3.53 | 3.57 | 2.75 | 4.13 |
| 24 | FEP | 10 | 25 | 55 | 3.95 | 2.94 | 3.46 | 3.51 | 3.28 | 4.04 |
| 25 | FEP | 16 | 44 | 82 | 4.03 | 2.79 | 3.61 | 3.77 | 2.95 | 4.36 |
| 26 | FEP | 31 | 18 | 9 | 4.17 | 2.65 | 3.71 | 3.91 | 3.14 | 4.50 |
| 27 | FEP | 2 | 34 | 0 | 3.76 | 2.51 | 3.00 | 3.05 | 2.85 | 3.92 |
| 28 | FEP | 8 | 27 | 56 | 4.00 | 2.85 | 3.44 | 3.71 | 2.67 | 4.27 |
| 29 | FEP | 9 | 86 | NA | 2.92 | 2.27 | 2.07 | 2.61 | 2.22 | 3.16 |
| 30 | FEP | 8 | 36 | 57 | 3.02 | 2.22 | 2.50 | 2.77 | 2.35 | 3.72 |
| 31 | FEP | 8 | 51 | 91 | 3.89 | 2.65 | 3.54 | 3.53 | 3.07 | 4.28 |
| 32 | FEP | 1 | 27 | 25 | 2.68 | 1.73 | 2.49 | 2.17 | NA | NA |
| 33 | FEP | 8 | 38 | 69 | 3.19 | 2.45 | 2.51 | 3.07 | 2.82 | 4.07 |
| 34 | HC | 3 | 12 | 33 | 4.30 | 2.90 | 3.41 | 3.74 | 2.68 | 4.13 |
| 35 | HC | 3 | 29 | 38 | 4.21 | 3.11 | 3.86 | 3.89 | 3.04 | 4.52 |
| 36 | HC | 1 | 28 | 22 | 4.40 | 2.59 | 3.92 | 3.69 | 2.91 | 4.08 |
| 37 | HC | 7 | 19 | 58 | 4.18 | 2.76 | 3.87 | 3.83 | 3.27 | 4.72 |
| 38 | HC | 0 | 28 | 43 | 4.73 | 3.44 | 4.14 | 4.27 | 3.68 | 5.17 |
| 39 | HC | 0 | 16 | 32 | 3.74 | 2.66 | 3.22 | 3.35 | 2.97 | 4.01 |
| 40 | HC | 3 | 10 | 34 | 4.24 | 3.63 | 3.94 | 3.94 | 3.76 | 4.23 |
| 41 | HC | 0 | 48 | 70 | 3.61 | 2.46 | 3.34 | 3.21 | 2.56 | 3.66 |
| 42 | HC | 0 | 4 | 3 | 4.72 | 3.06 | 4.30 | 4.26 | 3.21 | 4.68 |
| 43 | HC | 0 | 30 | 35 | 4.10 | 2.90 | 3.66 | 3.82 | 3.64 | 4.20 |
| 44 | HC | 7 | 61 | 54 | 3.51 | 2.62 | 2.98 | 3.17 | 2.80 | 3.81 |
| 45 | HC | 0 | 32 | 22 | 4.64 | 3.28 | 3.74 | 4.10 | 3.33 | 4.17 |
| 46 | HC | 0 | 29 | 35 | 4.25 | 2.56 | 3.69 | 3.57 | 3.23 | 4.12 |
| 47 | HC | 3 | 28 | 37 | 4.55 | 3.00 | 4.16 | 4.14 | 3.36 | 4.50 |
| 48 | HC | 1 | 59 | 40 | 3.42 | 2.10 | 3.24 | 2.81 | 2.16 | 3.71 |
| 49 | HC | 2 | 35 | 19 | 4.32 | 3.19 | 3.74 | 3.82 | 3.78 | 4.76 |
| 50 | CHR | 17 | 90 | 67 | 3.72 | 2.73 | 3.12 | 3.30 | 2.94 | 3.72 |
| 51 | FEP | 4 | 6 | 25 | 3.62 | 2.67 | 3.19 | 3.24 | 3.07 | 3.69 |
| 52 | CHR | 9 | 47 | 59 | 3.71 | 2.36 | 3.07 | 3.19 | 2.79 | 4.01 |
| 53 | CHR | 12 | 38 | 65 | 3.81 | 2.32 | 3.30 | 3.37 | 2.65 | 3.61 |
| 54 | FEP | 4 | 19 | 12 | 3.74 | 2.22 | 3.39 | 3.11 | 2.42 | 3.62 |
| 55 | CHR | 18 | 56 | 63 | 3.91 | 2.64 | 3.54 | 3.47 | 3.24 | 4.39 |
| 56 | CHR | 16 | 32 | NA | 3.59 | 2.44 | 3.07 | 2.78 | 2.71 | 3.50 |
| 57 | HC | 1 | 49 | 53 | 3.86 | 2.18 | 3.30 | 3.00 | 2.60 | 3.74 |
| 58 | FEP | 10 | 15 | 51 | 4.15 | 2.68 | 3.81 | 3.65 | 3.07 | 3.97 |
| 59 | CHR | 20 | 44 | 56 | 4.23 | 2.60 | 3.64 | 3.75 | 3.05 | 3.92 |
| 60 | FEP | 6 | 37 | 31 | 4.83 | 3.15 | 4.09 | 4.31 | 3.41 | 4.53 |
| 61 | FEP | 13 | 16 | 45 | 4.45 | 2.63 | 3.55 | 3.75 | 2.80 | 4.25 |
| 62 | FEP | 5 | 54 | 39 | 3.87 | 2.29 | 3.27 | 3.34 | 2.97 | 3.81 |
| 63 | HC | 0 | 49 | 35 | 4.53 | 2.93 | 3.99 | 3.84 | 3.39 | 4.47 |
| 64 | HC | 7 | 30 | 48 | 3.90 | 2.78 | 2.98 | 3.38 | 2.92 | 3.79 |
| 65 | CHR | 12 | 51 | 80 | 3.73 | 2.37 | 3.28 | 2.91 | 2.69 | 3.46 |
| 66 | CHR | 18 | 43 | 59 | 4.01 | 2.91 | 3.59 | 3.78 | 3.27 | 4.62 |
| 67 | CHR | 13 | 73 | 73 | 3.74 | 2.36 | 2.99 | 3.13 | 2.26 | 3.93 |
| 68 | HC | 3 | 18 | 31 | 3.91 | 2.40 | 3.33 | 3.27 | 2.74 | 3.98 |
| 69 | CHR | 2 | 26 | 35 | 4.59 | 3.49 | 4.03 | 4.18 | 3.69 | 4.99 |
| 70 | HC | 1 | 63 | 32 | 3.88 | 2.70 | 3.13 | 3.56 | 2.96 | 4.12 |
| 71 | CHR | 11 | 39 | NA | 3.50 | 1.53 | 3.15 | 2.70 | 2.59 | 3.45 |
| 72 | CHR | 9 | 40 | 35 | 3.21 | 1.96 | 2.59 | 2.70 | 2.22 | 3.31 |
| 73 | FEP | 2 | 0 | 5 | 3.85 | 2.83 | 3.34 | 3.38 | 3.56 | 3.84 |
| 74 | CHR | 4 | 15 | 49 | 3.49 | 2.45 | 3.18 | 3.01 | 2.76 | 3.78 |
| 75 | CHR | 5 | 50 | 21 | 4.51 | 3.01 | 3.77 | 3.85 | 3.42 | 4.57 |
| 76 | FEP | 5 | 38 | 29 | 3.76 | 2.55 | 3.06 | 3.03 | 2.71 | 3.94 |
| 77 | CHR | 1 | 12 | 26 | 3.99 | 2.49 | 3.45 | 3.50 | 3.01 | 3.79 |
| 78 | FEP | 7 | 38 | 12 | 3.54 | 2.44 | 3.06 | 2.99 | 2.84 | 4.34 |

| **Syntaxis** |  |  |  |  |  |  |  |  |  |  |  |  |  |  |  |
| --- | --- | --- | --- | --- | --- | --- | --- | --- | --- | --- | --- | --- | --- | --- | --- |
| MIXED BPnd BY ROI Group WITH **Stress_Scaleª** | | | |  |  |  |  |  |  |  |  |  |  |  |  |
| /CRITERIA=DFMETHOD(RESIDUAL) CIN(95) MXITER(100) MXSTEP(10) SCORING(1) SINGULAR(0.000000000001) | | | | | | | | |  |  |  |  |  |  |  |
| HCONVERGE(0.00000001, RELATIVE) LCONVERGE(0, ABSOLUTE) PCONVERGE(0, ABSOLUTE) | | | | | | | |  |  |  |  |  |  |  |  |
| /FIXED=ROI Group **Stress_Scaleª** **Stress_Scaleª***Group \| SSTYPE(3) | | | | | |  |  |  |  |  |  |  |  |  |  |
| /METHOD=ML | |  |  |  |  |  |  |  |  |  |  |  |  |  |  |
| /PRINT=SOLUTION TESTCOV | | |  |  |  |  |  |  |  |  |  |  |  |  |  |
| /RANDOM=INTERCEPT \| SUBJECT(id) COVTYPE(VC) | | | |  |  |  |  |  |  |  |  |  |  |  |  |
| /REPEATED=ROI \| SUBJECT(id) COVTYPE(DIAG) | | | |  |  |  |  |  |  |  |  |  |  |  |  |
|  |  |  |  |  |  |  |  |  |  |  |  |  |  |  |  |
| ªThe term **Stress_Scale** represents the selected scale for each model.  It should be replaced with Hassless score for model 1, TICS score for model 2 and the  squared-root transformed HDRS score for model 3. | | | | | | | | | | | | | | | |
